# Supplementary material for: Inferring directed spectral information flow between mixed-frequency time series
Source: Res Sq. 2025 Feb 28:rs.3.rs-4926819. Preprint. [Version 1] doi: 10.21203/rs.3.rs-4926819/v1 (PMC11888547; doi:10.21203/rs.3.rs-4926819/v1)
Supplement: 1 [file NIHPPRS4926819V1-supplement-1.pdf]

Supplementary Information

Inferring directed spectral information flow between mixed-frequency time series

Qiqi Xian and Zhe Sage Chen

Supplementary Note

Given two raw random time series $x(t)$ and $y(t)$, we may compute their cross-correlation function $R_{xy}(t)$. In the frequency domain, the Fourier transform of the cross-correlation function is written as: $\mathcal{F}\{R_{xy}(t)\} = S_{xy}(f) = X(f)Y^*(f)$. Let's further assume that two raw signals are filtered by two filters g and h, respectively; the two filtered signals are represented as: $\tilde{x}(t) = x(t) * g$ and $\tilde{y}(t) = y(t) * h$. Let $R_{\tilde{x}\tilde{y}}(t)$ denote the cross-correlation function between $\tilde{x}(t)$ and $\tilde{y}(t)$, then the Fourier transform of $R_{\tilde{x}\tilde{y}}(t)$ is written as $\mathcal{F}\{R_{\tilde{x}\tilde{y}}(t)\} = S_{\tilde{x}\tilde{y}}(f) = S_{xy}(f)G(f)H^*(f)$. If $x(t)$ and $y(t)$ are convolved with the same temporal low-pass filter, it may introduce interactions between instantaneous (i.e. zero-lag) and time-lagged relationships; in other words, there may be "leakage" of zero-lag correlation into time-lagged GC because of autocorrelation of $\tilde{x}(t)$ and $\tilde{y}(t)$ due to low-pass filtering. Therefore, the interpretation of results requires a careful examination of the observed signals. The standard VAR modeling can be modified to accommodate the instantaneous effect (e.g., Nuzzi et al., 2021; Desphande et al., 2010).

Down-sampling or subsampling requires a low-pass filter before decimation in the time domain, so that the sample frequency needs to be at least twice of the max frequency signal, otherwise the aliasing may emerge (i.e., high-frequency signal components will copy into the lower frequency band and be mistaken for lower frequencies). For instance, if $y(t)$ is represented by a discrete-time signal $y[n]$, with a down-sampling factor of *D*, we will obtain $y[Dn]$. In the frequency domain, the magnitude of Fourier spectrum of $y[Dn]$ is lowered by a factor of *D* compared to the magnitude of Fourier spectrum of $y[n]$. To avoid aliasing (especially when *D* is rather large), the cut-off frequency of the low-pass filter should be $0.5F_y$, where F_y denotes the sampling frequency of $y[Dn]$. Therefore, this combined operation (low-pass filter plus down-sampling) can be viewed as a special case of low-pass time-varying filter, which is not invariant to temporal shift. Consequently, down-sampling has an impact on the time-lagged causality.

Supplementary references

Nuzzi D, Stramaglia S, Javorka M, Marinazzo D, Porta A, Luca F. Extending the spectral decomposition of Granger causality to include instantaneous influences: application to the control mechanisms of heart rate variability. *Phil. Trans. R. Soc. A.***379**, 20200263 (2021).

Desphande G, Sathian K, Hu X. Assessing and compensating for zero-lag correlation effects in time-lagged Granger causality of fMRI. *IEEE Trans. Biomed. Eng.* **57,** 1446-1456 (2010).

Table S1. Summary of all computer simulation results.

**Condition**	**Driving frequency**	**Direction of information flow**	**Figure**
Bivariate system $\{X,Y\}$, $X(t)$ HF process, $Y(t)$ LF process			
$X \overset{f_x}{\rightarrow} Y$ (linear, unidirectional)	Recover f_x	Identify $X \rightarrow Y$ from lag-CC	Fig. S2
$X \overset{f_x}{\rightarrow} Y$ (linear, unidirectional)	Recover f_y	Identify $Y \rightarrow X$ from lag-CC	Fig. S2
$X \overset{f_x}{\rightarrow} Y$ and $Y \overset{f_y}{\rightarrow} X$ (linear, bidirectional, f_x>f_y)	Recover f_x but not necessarily f_y (depending on F_y)	Identify dominant direction (amplitude difference)	Fig. 2
$X \overset{f_x}{\rightarrow} Y$ and $Y \overset{f_y}{\rightarrow} X$ (bidirectional, nonlinear PAC)	Recover f_x and f_y	Identify dominant direction, recover the modulated (but not original) driving frequency	Fig. 3
$X \overset{f_x}{\rightarrow} Y$ and $Y \overset{f_y}{\rightarrow} X$ (bidirectional, sigmoidal amplitude coupling)	Recover f_x and f_y	Identify dominant direction and driving frequency	Fig. S4
$X \overset{f_x}{\rightarrow} Y$ and $Y \overset{f_y}{\rightarrow} X$ (bidirectional, sinusoidal amplitude coupling)	Recover f_x and f_y	Identify dominant direction and driving frequency	Fig. S4
Trivariate system $\{X_1, X_2, Y\}$ or $\{X, Y_1, Y_2\}$			
$X_1 \rightarrow X_2 \rightarrow Y$ (linear, unidirectional, chain system	Recover f_x	Misidentify $X_1 \rightarrow Y \vert X_2$ as putative GC pattern (lag-CC depends on the temporal shift of X_2)	Fig. 4
$X \rightarrow Y_1 \rightarrow Y_2$ (linear, unidirectional, chain system)	Recover f_x	Misidentify $X \rightarrow Y_2 \vert Y_1$ as putative GC pattern (lag-CC depends on the Y_1 term in the time or time-frequency domain)	Fig. S5
$X_1 \rightarrow Y$ and $X_2 \rightarrow Y$ (linear, unidirectional, parallel system)	Recover f_x	Identify $X_1 \rightarrow Y \vert X_2$ and $X_2 \rightarrow Y \vert X_1$, compared to $X_1 \rightarrow Y$ and $X_2 \rightarrow Y$	Fig. S6
two-species Logistic model $\{X,Y\}$			
Nonlinear, uni- or bidirectional	n/a	Identify the directionality of information flow	Fig. S7
coupled Rössler-Lorenz system $\{\boldsymbol{X},\boldsymbol{Y}\}$			
Nonlinear, unidirectional	n/a	Identify the directionality of information flow	Fig. S8

Table S2. Summary results of qualitative comparison between MF-TFCCA and MF-VAR methods. In this table, the down-sampling factor is 5 and sampling frequency ratio is 5:1. False positives are marked in red.

Condition	MF-TFCCA detected information flow	MF-VAR detected information flow
$X \overset{f_x}{\rightarrow} Y$ (linear, unidirectional)	$X \rightarrow Y$ (Fig. S1)	$X \rightarrow Y$
$Y \overset{f_y}{\rightarrow} X$ (linear, unidirectional)	$Y \rightarrow X$ (Fig. S1)	$X \rightarrow Y$ and $Y \rightarrow X$
$X \overset{f_x}{\rightarrow} Y$ and $Y \overset{f_y}{\rightarrow} X$ (linear, bidirectional)	$X \rightarrow Y$ and $Y \rightarrow X$ (Fig. 2)	None
$X_1 \rightarrow X_2 \rightarrow Y$ (linear, unidirectional, chain system	Partially observed: $X_1 \rightarrow Y$ Fully observed: *reduced* $X_1 \dashrightarrow Y\|X_2$ and $X_2 \rightarrow Y\|X_1$ (Fig. 4)	Partially observed: $X_1 \rightarrow Y$ Fully observed: $X_1 \rightarrow Y\|X_2$ and $X_2 \rightarrow Y\|X_1$
$X \rightarrow Y_1 \rightarrow Y_2$ (linear, unidirectional, chain system)	Partially observed: $X \rightarrow Y_2$ Fully observed: *reduced* $X \dashrightarrow Y_2\|Y_1$ (Fig. S5)	Partially observed: $X \rightarrow Y_2$ Fully observed: $X \rightarrow Y_1\|Y_2$ and $X \rightarrow Y_2\|Y_1$
$X_1 \rightarrow Y$ and $X_2 \rightarrow Y$ (linear, unidirectional, parallel system)	Partially and fully observed: $X_1 \rightarrow Y$ and $X_2 \rightarrow Y$ (Fig. S6)	Partially observed: $X_1 \rightarrow Y$ and $X_2 \rightarrow Y$ Fully observed: $X_1 \rightarrow Y$, $X_2 \rightarrow Y$ and $X_1 \leftrightarrow X_2$
$X \overset{f_x}{\rightarrow} Y$ and $Y \overset{f_y}{\rightarrow} X$ (nonlinear PAC, bidirectional)	$X \rightarrow Y$ and $Y \rightarrow X$ (Fig. 3)	None

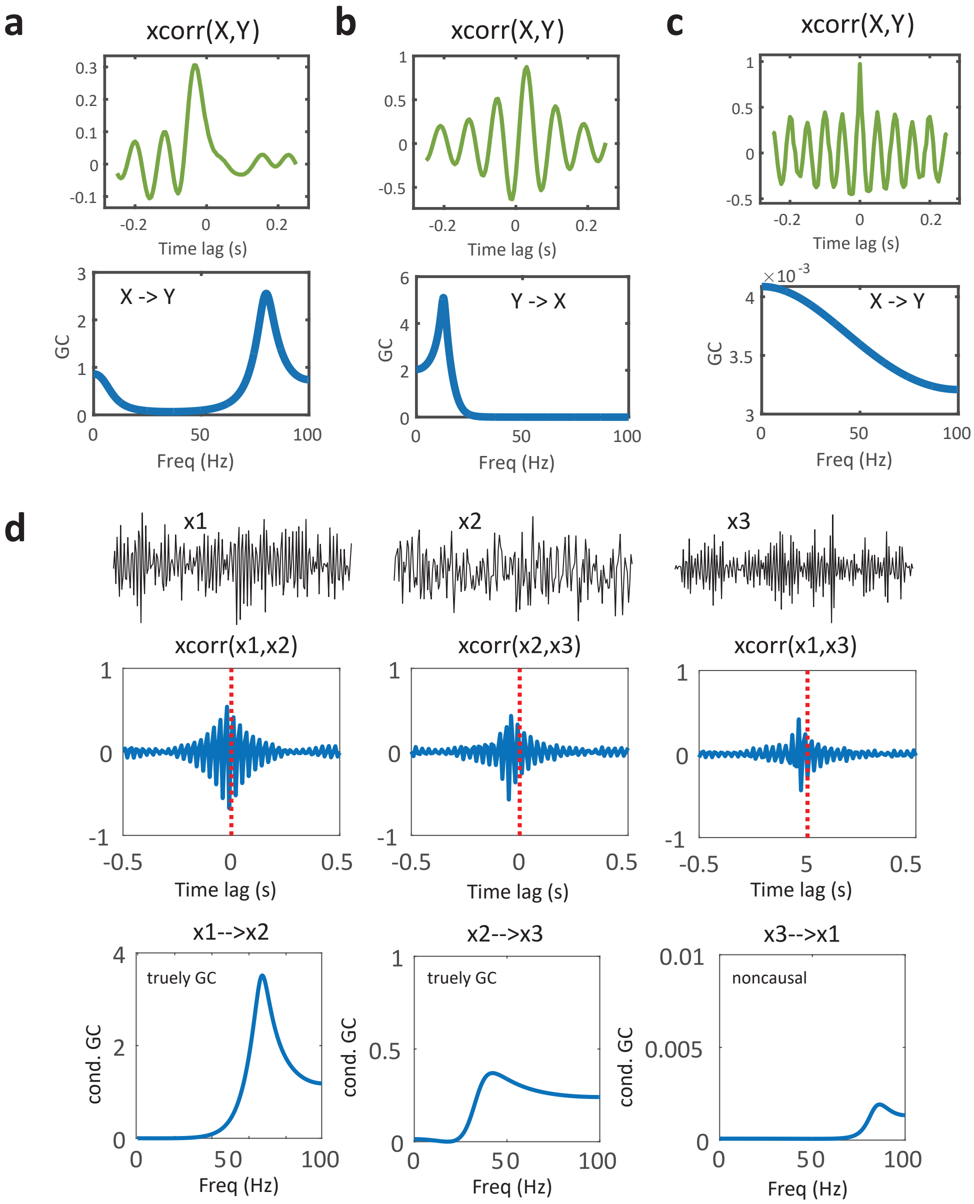

Figure S1. Lagged cross-correlation (xcorr) between two random variables is neither sufficient nor necessary condition for Granger causality (GC). Here all time-series have the same sampling frequency.

(a) X Granger causes Y, with a clear asymmetric xcorr profile.

(b) X Granger causes Y, but the xcorr profile is nearly symmetric.

(c) X and Y has a pronounced xcorr profile, but there is no statistically significant GC.

(d) *Top:* Snapshots of time series $\{x_1, x_2, x_3\}$ generated from a VAR(3) based on REF[54]; *Middle:* xcorr profiles; *Bottom:* ground truth conditional SGC (x_1 Granger causes x_2 and x_2 Granger causes x_3). In this case, there is no a clear indication of GC from xcorr.

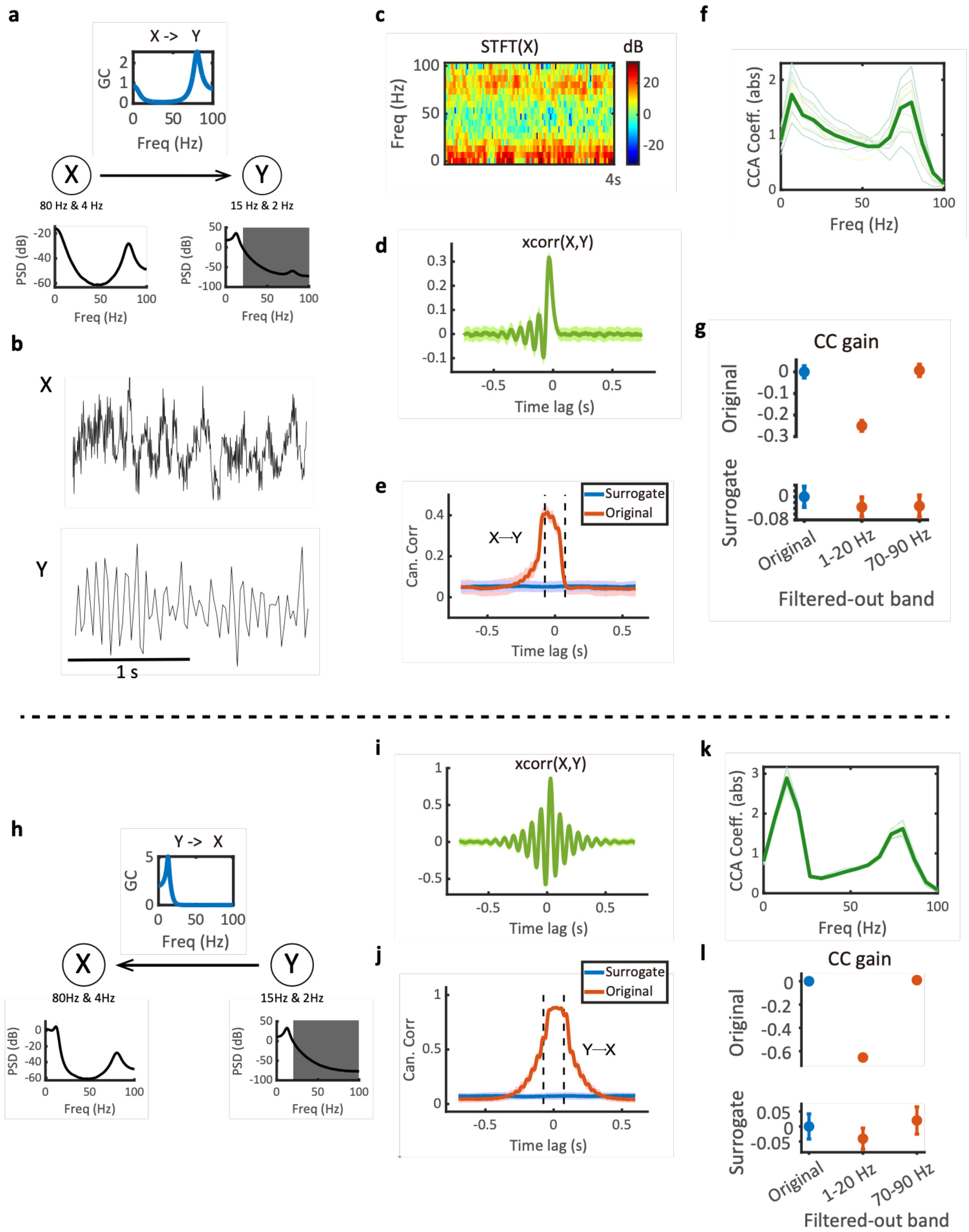

Figure S2. Computer simulation results on bivariate unidirectional Granger causality (GC).

(a) Illustration of the unidirectional GC ($X \overset{f_x}{\to} Y$) at both low frequency f_x = 4 Hz and high frequency f_x = 80 Hz and the power spectral density (PSD) of two time series. X has a low-frequency component and a high-frequency component, and Y has two resonant frequencies in the low-frequency band. The shaded area in the PSD denotes the filtered-out frequency band above the cut-off frequency (20 Hz) of down-sampled Y.

(b) The 2-s snapshots of bivariate time series.

(c) The 4-s snapshot of short-time Fourier transform (STFT) of $X(t)$.

(d) Lagged cross-correlation profile generated from xcorr(X,Y).

(e) Lag-CC profile generated from linear MF-TFCCA, which detected directed information flow from X to Y. Red trace denotes the estimate derived from the data, and blue trace denotes the estimate derived from the surrogate data. Shade areas denote 95% confidence intervals (n=100). The significant CC profile located beyond the left dashed line suggests causal information from X to Y.

(f) The MF-TFCCA coefficients computed at a negative lag show a peak in the low-frequency range and another peak in the high-frequency range, corresponding to the two resonant frequencies of $X(t)$: 4 Hz and 80 Hz. The bold red line indicates the trial-averaged coefficients, and the light lines indicate the coefficients estimated from single trials.

(g) The CC gain (ΔCC relative to the original unfiltered setting) by band-stop filtering of relevant frequency band in the HF signal X. A negative gain suggests greater importance of the putative driving frequency (i.e., 4 Hz) within the filter-out band, whereas a zero gain suggests negligible change in CC of the other putative driving frequency (e.g., 80 Hz).

(h) Illustration of the unidirectional GC ($Y \overset{f_y}{\to} X$) at only low frequency f_y = 15 Hz and the PSD of two time series. The shaded area in the PSD denotes the filtered-out frequency band above the cut-off frequency (20 Hz) of down-sampled Y.

(i) Lagged cross-correlation profile generated from xcorr(X,Y).

(j) Lag-CC profile generated from linear MF-TFCCA recovered a directed information flow from Y to X.

(k) The CCA coefficients (in absolute value) computed from MF-TFCCA at a positive lag showed a higher peak in 15 Hz than in 80 Hz. Despite down-sampling, the driving frequency of X was preserved.

(l) The relative CC gain (ΔCC relative to the original unfiltered setting) by band-stop filtering of relevant frequency band in the HF signal Y. A negative gain suggests greater importance of the putative driving frequency (i.e., 15 Hz) within the filter-out band, whereas a zero gain suggests negligible change in CC induced by the other putative driving frequency (i.e., 80 Hz).

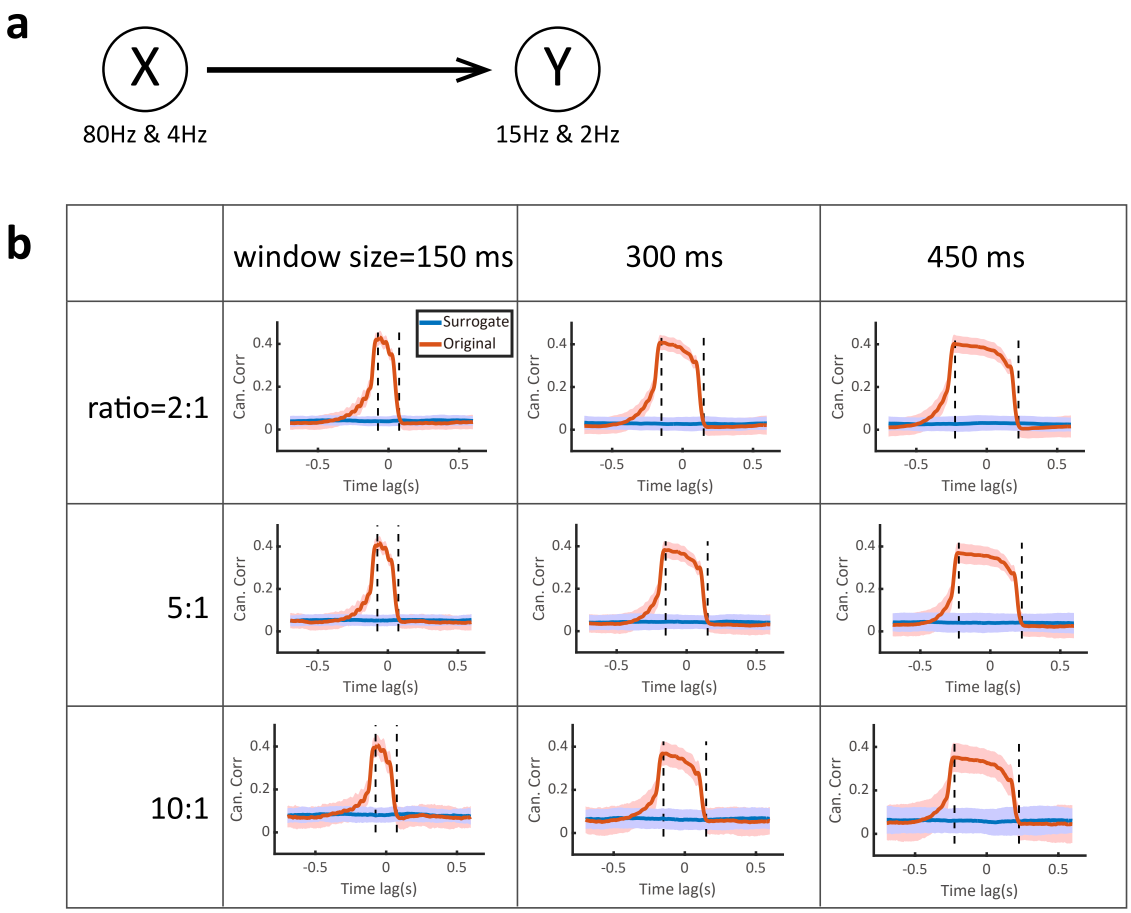

Figure S3. Comparison of estimated lag-CC profiles from MF-TFCCA with various sampling frequency ratios $\frac{F_x}{F_y}$ and STFT window sizes.

- **(a)** Illustration of the unidirectional GC system (same as **Fig. S2a**).
- **(b)** The estimated lagged cross-correlation profile under different conditions with various sampling frequency ratios and window sizes in STFT. The significant CC profile located beyond the left dashed line suggests causal information from X to Y. Note that the window size determines the detection resolution (i.e., the duration between two vertical dashed lines).

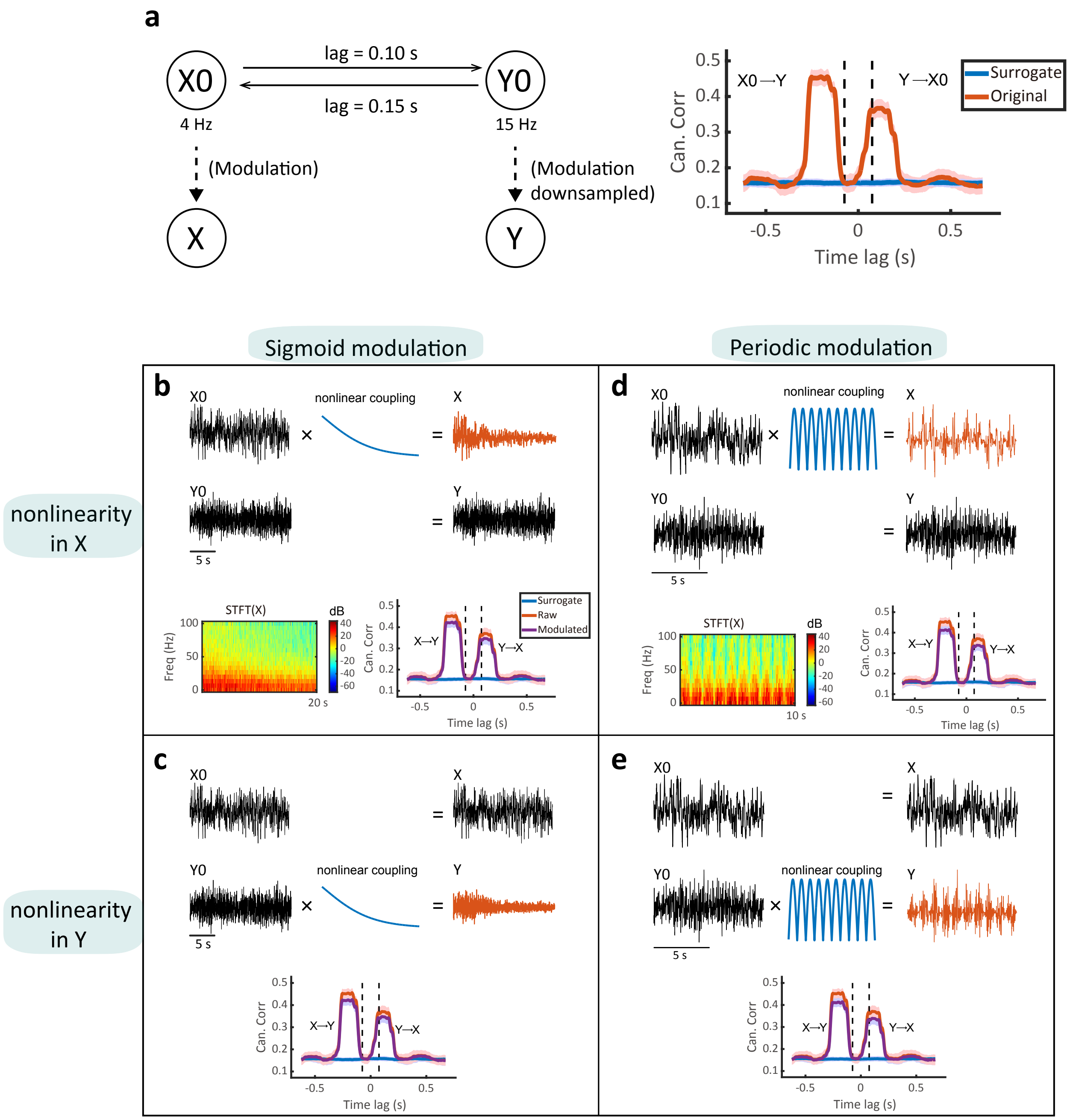

Figure S4. Computer simulation results on bivariate nonlinear Granger causality (GC) with amplitude modulation.

- **(a)** The original bivariate linear GC system, with $X_0 \overset{f_x}{\to} Y_0$ at f_x = 4 Hz and $Y_0 \overset{f_y}{\to} X_0$ at f_y = 15 Hz. The nonlinear causal system variables X and Y are generated by modulating the amplitude X_0 or Y_0. MF-TFCCA recovered the bidirectional causal structure between X_0 and Y_0.
- **(b)** Nonlinear causal system with sigmoid amplitude modulation. *Top:* the modulated signal X was produced by the raw signal X_0 multiplying a sigmoid function, where Y remained intact. *Bottom left:* the STFT

spectrum of the modulated X shows the decay in the power of X. *Bottom right*: the lag-CC curve revealed bidirectional nonlinear causal relationship between X and Y.

(c) Similar to panel **b**, but the sigmoid modulation was applied to Y. Similar results were found.

(d) A nonlinear GC system with periodic amplitude modulation. The modulated signal X was produced by the raw signal X_0 multiplying a sine function, where Y remained intact. The STFT spectrum of the modulated X showed a periodic pattern. The lag-CC curve also revealed bidirectional nonlinear causal relationship between X and Y.

(e) Similar to panel **d**, but the periodic modulation was applied to Y. Similar results were found.

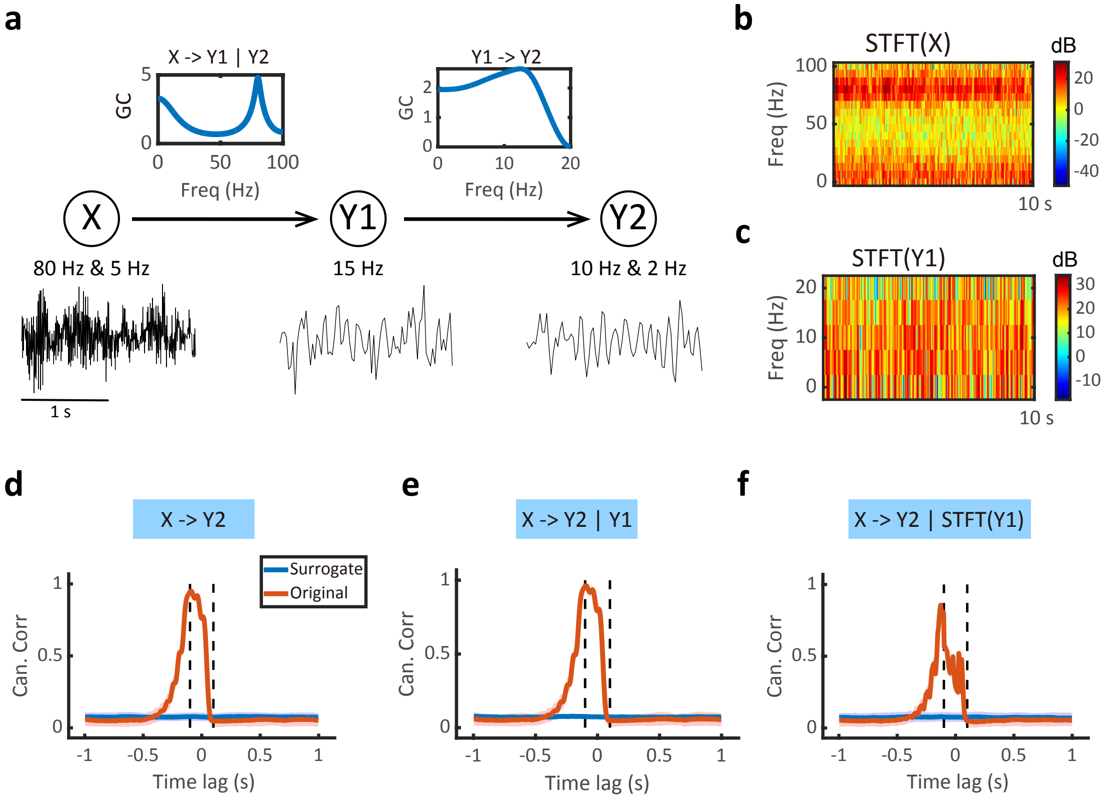

Figure S5. Computer simulation results on trivariate Granger causality (GC) in a chain system with the intermediate variable in low sampling rate.

- **(a)** Illustration of the chain system $X \rightarrow Y_1 \rightarrow Y_2$ as well as the conditional GC profiles. Note that Y_1 and Y_2 have low sampling rates (40 Hz), whereas X has a high sampling rate (200 Hz). The system is similar to the chain system in **Fig. 4** except that Y_1 here was down-sampled from X_2 in **Fig. 4**.
- **(b)** STFT spectrogram of X.
- **(c)** STFT spectrogram of Y_1. Note that in order to match the length of STFT(X), the overlap of STFT window was different, resulting in different temporal resolution.
- **(d)** Lag-CC profile generated from MF-TFCCA without considering Y_1, showing directed information flow from X to Y_2. Red trace denotes the estimate derived from the original data, and blue trace denotes the estimate derived from the surrogate data. Shade areas denote 95% confidence intervals (n=100 trials).
- **(e)** Similar to panel **d**, but the lag-CC profile was computed based on partializing Y_1 in the time domain. The lag-CC magnitude was not reduced by partializing the time series Y_1.
- **(f)** Similar to panel **d**, but the lag-CC profile was computed based on partializing the spectrum $\mathrm{STFT}(Y_1)$ in the time-frequency domain. $\mathrm{STFT}(Y_1)$ was computed using the same window size as $\mathrm{STFT}(X)$ and with the same overlap of the moving window as the sampling interval of Y. Note that the lag-CC magnitude decreased after partializing Y_1 spectrum, suggesting a chain structure in this system. However, the residue was still large due to the information loss of down-sampling Y_1.

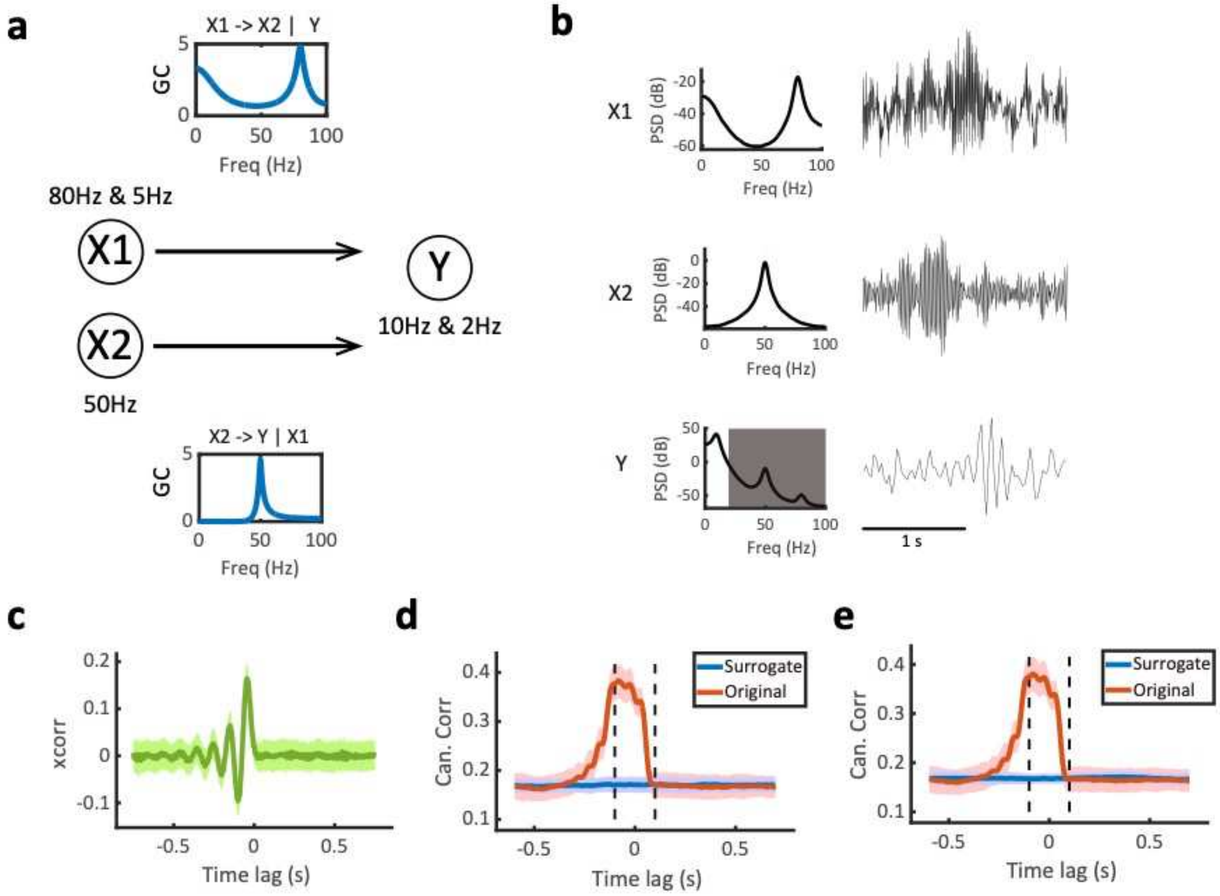

Figure S6. Computer simulation results on trivariate Granger causality (GC) in a parallel system.

(a) Illustration of the parallel system $X_1 \to Y$ and $X_2 \to Y$ as well as the conditional GC profiles.

(b) 2-s snapshots of trivariate time series and their power spectral density (PSD). Note that X_1 and X_2 have high sampling rates (200 Hz), whereas Y has a low sampling rate (40 Hz). The shaded area in the PSD denotes the filtered-out frequency band above the cut-off frequency (20 Hz) of down-sampled Y.

(c) The lagged cross-correlation profile generated from xcorr(X_1,Y) without considering X_2.

(d) The lag-CC profile generated from MF-TFCCA without partialization of X_2, showing a directed information flow from X_1 to Y. Red trace denotes the estimate derived from the original data, and blue trace denotes the estimate derived from the surrogate data. Shade areas denote 95% confidence intervals (n=100 trials).

(e) The lag-CC profile generated from MF-TFCCA with partialization of X_2, showing a similar directed information flow from X_1 to Y; namely, the driving of $X_2 \to Y$ did not affect detecting $X_1 \to Y$.

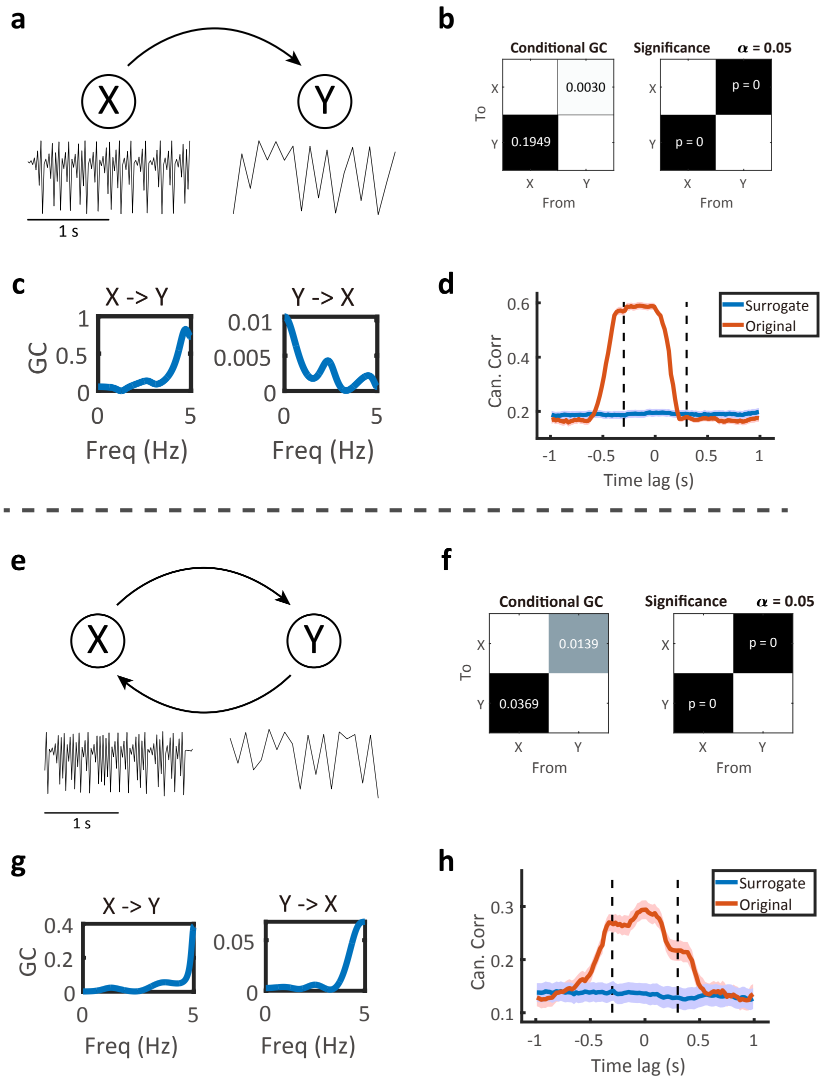

Figure S7. Computer simulation results on the two-species Logistic model.

- **(a)** Illustration of the unidirectional system $X \rightarrow Y$, where Y has a lower sampling rate than X by a factor of 5.
- **(b)** Time-domain GC test showing GC value (left) and p-value (right) under a significance level of 0.05.
- **(c)** SGC profiles in the frequency domain. A dominant SGC coefficient was found in the $X \rightarrow Y$ direction.
- **(d)** The lag-CC profile generated from MF-TFCCA discovered a directed information flow from X to Y. Red trace denotes the estimate derived from the original data, and blue trace denotes the estimate derived from the surrogate data. Shade areas denote 95% confidence intervals. The significant CC profile located beyond the left dashed line suggests causal information from X to Y.
- **(e)** Illustration of the bidirectional system $X \leftrightarrow Y$.
- **(f)** Time-domain GC test showing GC value (left) and p-value (right) under a significance level of 0.05.
- **(g)** Greater SGC coefficients were found in the $X \rightarrow Y$ direction than the $Y \rightarrow X$ direction.
- **(h)** The lag-CC profile generated from MF-TFCCA discovered a bidirectional information flow, as suggested by the significant CC profile located beyond both left and right vertical dashed lines.

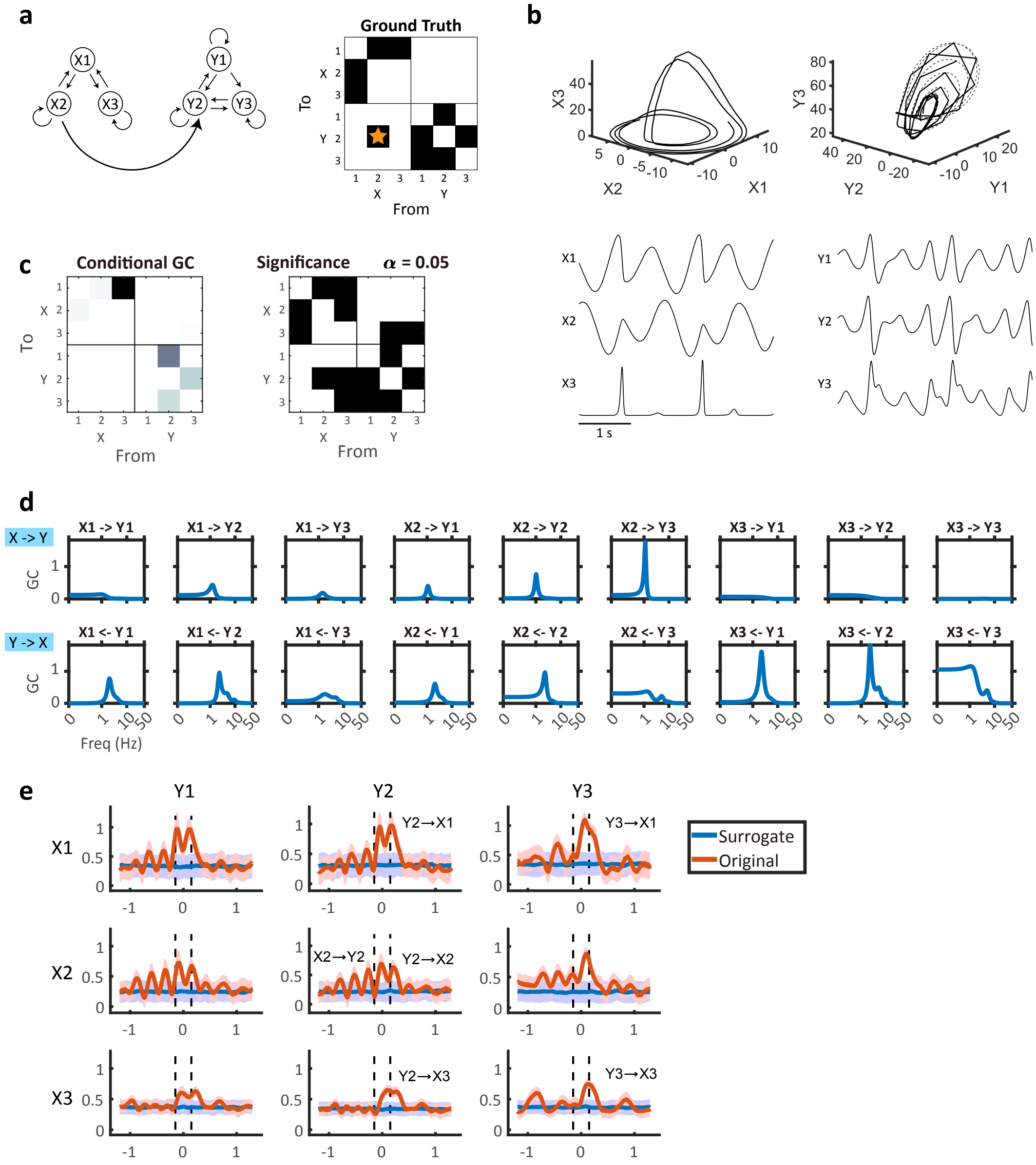

Figure S8. Computer simulation results on the coupled Rössler-Lorenz system.

- **(a)** Illustration of the true causal relationship of all 6 observed variables $\boldsymbol{X} = \{x_1, x_2, x_3\}$ and $\boldsymbol{Y} = \{y_1, y_2, y_3\}$. The black entries denote the statistical dependency between variables in terms of system dynamics. The black entry with "★" symbol illustrates the nonlinear coupling $x_2 \rightarrow y_2$ between two systems.
- **(b)** Snapshots of state-space portraits and time series for HF signals $\boldsymbol{X}$ and LF signals $\boldsymbol{Y}$.
- **(c)** Standard GC test in the time domain based on observed time series with equal sampling frequency, which shows the GC values (left) and statistical significance (right).
- **(d)** Estimated SGC in the frequency domain based on observed time series with equal sampling frequency. Note that the frequency axis is shown in the log scale.
- **(e)** The lag-CC profiles for all pairwise MF signals in MF-TFCCA (based on real-valued STFT spectrum).

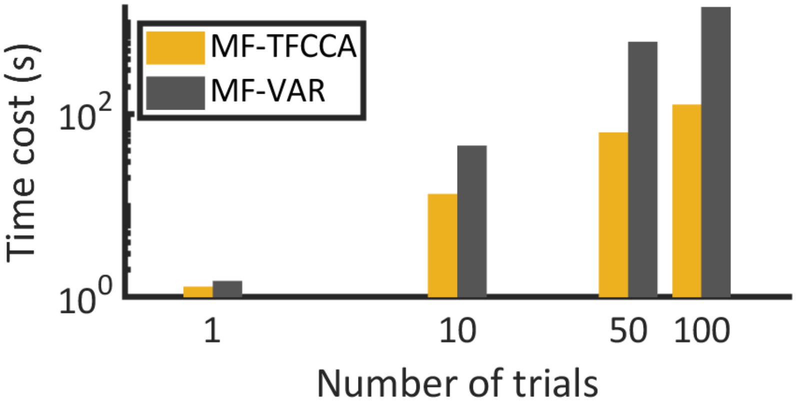

Figure S9. Benchmark comparison between MF-TFCCA and MF-VAR on computational cost. In this illustration, we used a simple bivariate VAR(1) system setup with unidirectional GC and a down-sampling factor of 5. Notice that the CPU time cost increased nearly quadratically for MF-VAR in terms of number of trials for a single-lag computation, whereas increased linearly for MF-TFCCA. Note that the plot is in log-log scale. In computer simulations, each trial contained 4000 samples of the HF time series, and 800 samples of the LF time series. The computational cost also included computation of 100 repetitions of surrogate data.

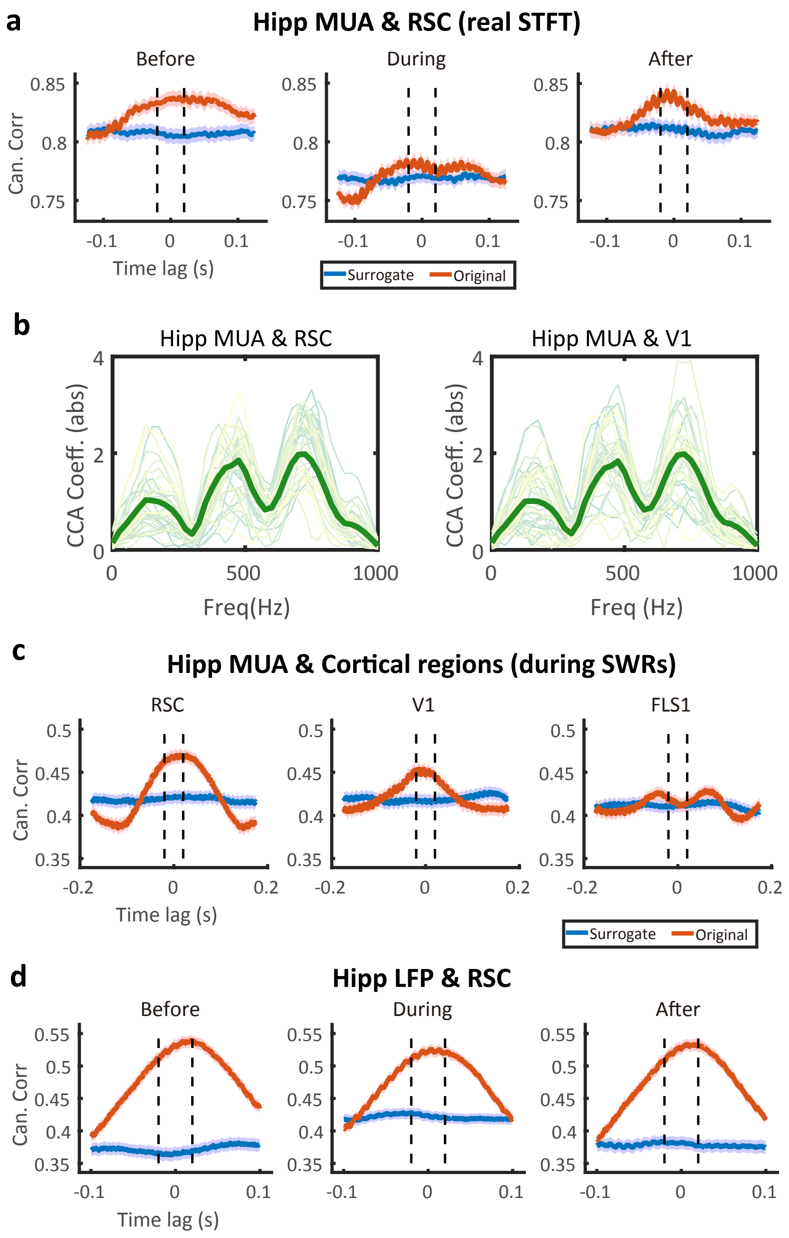

Figure S10. Inferred directed information flow in mouse hippocampal-neocortical recordings.

- **(a)** The lag-CC profiles for high-sampled hippocampal MUA and low-sampled RSC activity before, during, and after ripple events. The original CC values (red) were compared against the surrogate data estimates (blue).
- **(b)** CCA coefficients (in absolute value) between hippocampal MUA and RSC activity (left) as well as between hippocampal MUA and V1 activity (right). Thick red lines denote the trial average from single trials (thin colored lines). Multiple peaks were found, revealing the driving frequencies of MUA.
- **(c)** The lag-CC profiles for high-sampled hippocampal MUA and low-sampled RSC, V1, and FLS1 activities during sharp-wave ripples (SWRs).
- **(d)** The lag-CC profiles for high-sampled hippocampal LFP and low-sampled RSC activity before, during, and after ripple events. Similar to the hippocampal MUA result, a bidirectional information flow was identified.
